# Epigenomics of mammary gland development

**DOI:** 10.1186/s13058-018-1031-x

**Published:** 2018-09-03

**Authors:** Holly Holliday, Laura A. Baker, Simon R. Junankar, Susan J. Clark, Alexander Swarbrick

**Affiliations:** 1 The Kinghorn Cancer Centre, Cancer Research Division, Garvan Institute of Medical Research, Darlinghurst, NSW 2010 Australia; 2 St Vincent’s Clinical School, Faculty of Medicine, UNSW, Darlinghurst, NSW 2010 Australia; 3 Epigenetics Research Program, Genomics and Epigenetics Division, Garvan Institute of Medical Research, Darlinghurst, NSW 2010 Australia

**Keywords:** Mammary development, Breast cancer, Stem cell, Differentiation, Epithelial, Epigenetics, Chromatin, Methylation, Histone

## Abstract

Differentiation of stem cells into highly specialised cells requires gene expression changes brought about by remodelling of the chromatin architecture. During this lineage-commitment process, the majority of DNA needs to be packaged into inactive heterochromatin, allowing only a subset of regulatory elements to remain open and functionally required genes to be expressed. Epigenetic mechanisms such as DNA methylation, post-translational modifications to histone tails, and nucleosome positioning all potentially contribute to the changes in higher order chromatin structure during differentiation. The mammary gland is a particularly useful model to study these complex epigenetic processes since the majority of its development is postnatal, the gland is easily accessible, and development occurs in a highly reproducible manner. Inappropriate epigenetic remodelling can also drive tumourigenesis; thus, insights into epigenetic remodelling during mammary gland development advance our understanding of breast cancer aetiology. We review the current literature surrounding DNA methylation and histone modifications in the developing mammary gland and its implications for breast cancer.

## Background

### Lineage commitment in the mammary gland

The mammary gland is a dynamic tissue with rapid changes in tissue architecture occurring throughout the lifetime of the mammal in response to hormonal cues (reviewed in [1, 2]). The gland is comprised of an epithelial ductal tree embedded within a stromal fat pad comprised of a variety of cell types including adipocytes, fibroblasts, immune cells, lymphatic cells, and vascular cells that interact with each other to maintain a functional organ [2]. At birth, the gland contains a rudimentary ductal structure. The presence of oestrogen at puberty causes the ducts to undergo branching morphogenesis, generating a ductal tree that invades the stromal fat pad. Ductal elongation is driven by proliferation of cap cells located at the tips of the terminal end buds (TEBs) [1, 2]. During pregnancy and lactation, progesterone and prolactin cause extensive secondary and tertiary side branching and the formation of alveolar units that produce and secrete milk. Weaning of the offspring initiates the process of involution, which essentially remodels the mammary gland back to the virgin state [1, 2].

**Fig. 1:**
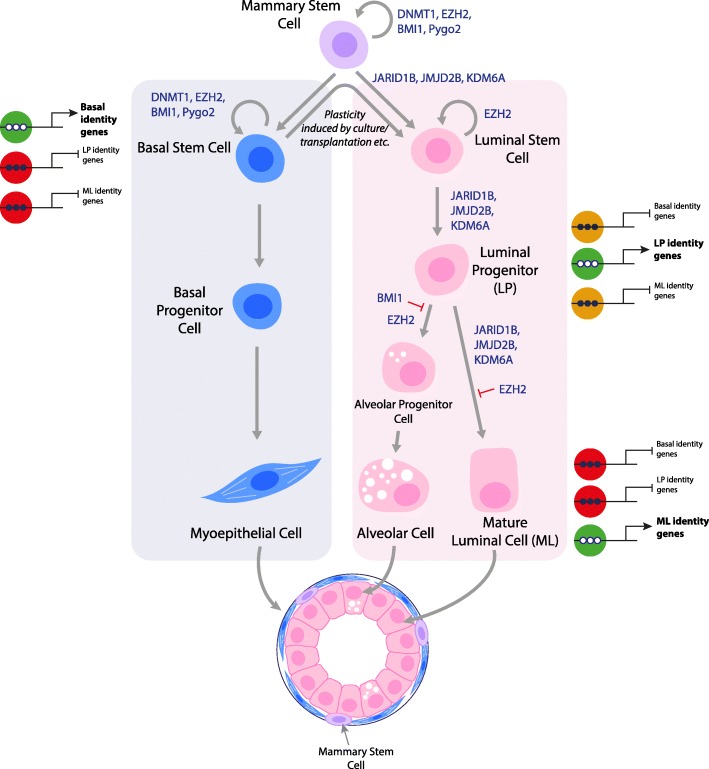
Model for the epigenetic regulation of lineage commitment within the mammary gland epithelium. Mammary stem cells (MaSC) located in the basal compartment can give rise to both the myoepithelial and luminal/alveolar lineages. Bipotent MaSCs may only be present during embryonic development and when basal stem cells are taken out of their physiological context (for example, upon dissociation and transplantation or culture). Epigenetic modifiers that have been shown to be involved in cell fate decisions are shown. Cells within the basal compartment (including stem, progenitor, and differentiated myoepithelial cells) have an epigenetic landscape that allows basal identity genes to be turned on and luminal identity genes to be turned off. Luminal progenitor cells have intermediate epigenetic features between basal and mature luminal cells. Mature luminal cells have repressive epigenetic features in basal and luminal progenitor identity genes and active epigenetic features in mature luminal identity genes. Red = repressed, orange = poised, green = active chromatin modifications. Open circles represent unmethylated promoter DNA; closed circles represent methylated promoter DNA

The mammary ductal epithelium is comprised of two main cell lineages: the inner luminal population containing ductal and alveolar cells, and the outer basal population containing myoepithelial cells (Fig. 1). The basal population is enriched for cells capable of self-renewal and multi-lineage differentiation upon serial transplantation into cleared mammary fat pads. These cells, known as mammary stem cells (MaSCs) [3, 4], lack unique cell surface markers, complicating their purification from the bulk basal cell population (referred to as ‘basal’). The luminal compartment contains proliferative luminal progenitor and mature luminal cells. During pregnancy and lactation, luminal progenitors differentiate into alveolar cells via alveolar progenitors. Transplantation experiments support a model whereby a bipotent MaSC at the apex of a differentiation hierarchy gives rise to both myoepithelial and luminal lineages [1]. The existence of bipotent MaSCs under physiological conditions is debated, with various lineage-tracing experiments yielding irreconcilable results. Some groups have found that adult basal cells give rise to both mature luminal and basal cells [5, 6]. Other groups have found that bipotent MaSCs only exist during embryonic development and that basal and luminal lineages of the adult gland are maintained by distinct pools of unipotent stem cells [7–10] (Fig. 1). The different conclusions may be due to reliance on different genetic reporters that mark distinct cell populations with discrete differentiation potentials.

It is increasingly accepted that cellular differentiation is not unidirectional and that ‘terminally differentiated’ cells may exhibit plasticity under certain conditions of stress, injury, or experimental stimuli [11]. Indeed, differentiated myoepithelial and luminal cells have been shown to adopt stem-like properties when cultured ex vivo [12]. The molecular mechanisms underlying this cellular plasticity are largely unknown.

### Epigenetics and development

**Fig. 2:**
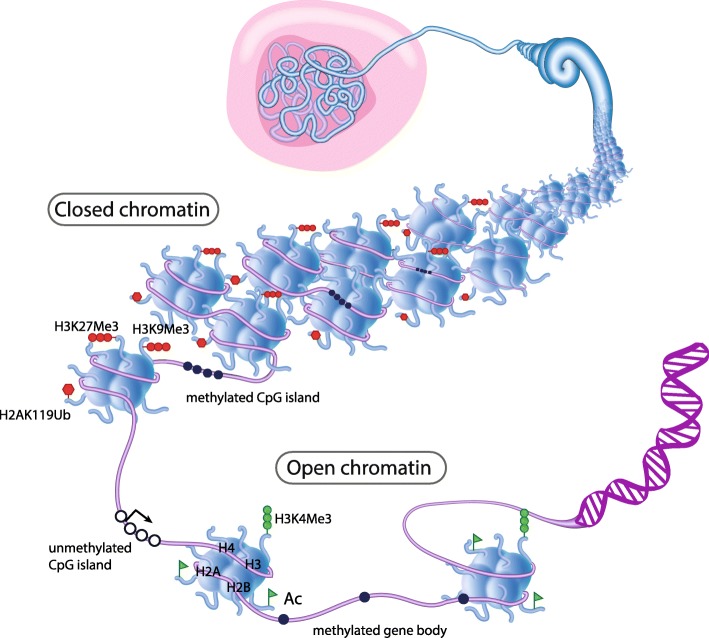
Epigenetic regulation of chromatin structure. The majority of DNA is packaged into inactive heterochromatin which is marked by repressive histone marks H3K27Me3, H3K9Me3, H2AK119Ub, and methylated CpG islands (closed circles) in gene promoters. Regions of open chromatin allow for activation of gene transcription and are marked by active histone marks H3K4Me3 and general histone acetylation as well as unmethylated CpG islands (open circles) in gene promoters and methylation of gene bodies. Promoter-enhancer looping (not shown) is another layer of epigenetic regulation of gene expression

Extensive changes in gene expression are required for a stem cell to undergo lineage commitment and functional differentiation during development. Changes in gene expression are associated with heritable epigenetic modifications to DNA and chromatin without changes to the DNA sequence. There are several layers of epigenetic regulation involved in the moderation of gene expression, including DNA methylation, post-translational modification to histone tails, chromatin remodelling, and higher order chromosome organisation (Fig. 2). DNA methylation is central to transcriptional repression, genomic imprinting, X-chromosome inactivation, and suppression of repetitive genomic elements [13, 14]. The basic chromatin subunit is the nucleosome, which is made up of ~ 147 nucleotides of DNA wrapped around a core histone octamer made up of two of each of the histone proteins H2A, H2B, H3, and H4 [15] (Fig. 2). Protruding N-terminal histone tails are subject to covalent post-translational modifications including acetylation, phosphorylation, ubiquitination, and methylation. The resulting ‘histone code’ ultimately influences gene transcription through multiple mechanisms (reviewed in [16]). The organisation and positioning of nucleosomes also determines which regions of the genome are active or inactive. Chromatin remodellers use ATP hydrolysis to move, destabilise, eject, or restructure nucleosomes to change chromatin accessibility and gene transcription [17].

Epigenetic regulation of differentiation in embryonic stem (ES) cells and the haematopoietic system is well characterised [18, 19]; however, far less is known about the epigenetic mechanisms underlying self-renewal and differentiation of tissue-specific epithelial stem and progenitor cells. Lineage-specific epigenetic programmes can become deregulated and result in oncogenesis [20]. Understanding these processes under physiological circumstances provides insights into how these complex programmes are altered during carcinogenesis.

## DNA methylation

The majority of CpG dinucleotides in the mammalian genome are methylated with the exception of CG-dense regions located around transcriptional start sites, known as CpG islands. These occur in approximately 70% of annotated gene promoters [14]. During development, DNA methyltransferase enzymes (DNMT1, DNMT3A, and DNMT3B) deposit and maintain methylation in a small subset of CpG island promoters causing transcriptional repression [21]. Conversely, methylation in gene bodies correlates with transcriptional activation [22] (Fig. 2). Cellular differentiation is accompanied by progressive gain of CpG island methylation resulting in silencing of developmental and non-related lineage-specific genes [23]. Demethylation of tissue-specific genes also defines cellular identity and is mediated by ten-eleven translocation (Tet) methylcytosine dioxygenases (Tet1, Tet2, Tet3) [23, 24].

### DNA methylation patterns define mammary epithelial cell identity

Mammary epithelial identity is, in part, shaped by the DNA methylation landscape, which varies between cell types [25, 26]. For example, methylation profiles from mammary epithelial cells are more similar to skin cells than blood cells, in keeping with the ectodermal origin of mammary and skin epithelial cells [25].

There have been numerous efforts to analyse DNA methylation patterns of stem/progenitor cells and differentiated luminal and myoepithelial cells in mice and humans [26–30]. Stem/progenitor cells isolated from human mammary glands are hypomethylated compared with differentiated luminal and myoepithelial cell types. This suggests that DNA methylation increases as the cells undergo lineage restriction, complementing work in other stem cell populations [31]. Genes that are hypomethylated and highly expressed in stem/progenitor cells include transcription factors with known roles in stem cell maintenance (e.g. HOXA1 and TCF7L1) [27]. Conversely, genes with promoter methylation (silenced) in stem/progenitor cells and gene body methylation (activated) in mature luminal cells include luminal-driving transcription factors (e.g. GATA3). This implies that DNA methylation is important in regulating the expression of lineage-specific transcription factors [28]. Comprehensive DNA methylation profiling of mouse mammary epithelial subpopulations has revealed similar patterns; when compared to basal cells, luminal cells have hypermethylation and silencing of stem and basal cell-related genes (e.g. *Angptl2* and *Krt5*) and hypomethylation and activation of epithelial differentiation genes (e.g. *Elf5*, *Cldn4*, and *Krt8*) [25, 26, 30]. Thus, DNA methylation is important for controlling expression of transcription factors that define basal cells and luminal populations.

Murine studies have been particularly useful in determining the effects of ageing and pregnancy on DNA methylation in mammary epithelial cells [25, 26]. Pregnancy induces changes to the DNA methylome of both basal and luminal populations that persist throughout life [25, 26]. Genes involved in lactation and involution become hypomethylated in response to the pregnancy and are primed to respond robustly to subsequent pregnancies [26]. An epigenetic memory of pregnancy may explain the protective effect of early pregnancy on breast cancer risk in humans later in life [32].

### DNMT1 maintains mammary stem/progenitor cells

DNMT1 has high affinity for hemimethylated DNA and is responsible for restoring the original methylation pattern present before DNA replication [21]. Its activity is required for the maintenance of adult stem cells including MaSCs [33] (Table 1). Expression of DNMT1 is similar in basal and luminal cells and increases during pregnancy [33]. Mammary-specific deletion of DNMT1 in mice severely effects TEB development and ductal elongation, results in fewer proliferative Ki67^+^ mammary epithelial cells, and mammary cells have reduced mammosphere-forming capacity when cultured ex vivo [33]. Correspondingly, inhibition of DNMT activity by administering 5-azacitidine (5-AzaC) to mice decreases Cyclin D1 expression and an overall reduction in mammary cell numbers [25]. Taken together, DNMT1-mediated DNA methylation is essential for maintenance of stem/progenitor cells in the mammary gland.

**Table 1:** Summary of epigenetic modulating proteins with known roles in mammary gland development

Protein complex or family	Members		Modification	Activating/repressive	Role in MG development	Reference
DNA methyltransferase	DNMT1, DNMT3a, DNMT3b		DNA methylation	Repressive at promoters, activating in gene bodies	DNMT1 maintains stem/progenitor cells	[33]
Polycomb repressive complex 2 (PRC2)	SUZ2, EED, RBBP4 or RBBP7 and EZH2 or EZH1		H3K27 trimethylation	Repressive	EZH2 maintains stem/progenitor cells, restricts mature luminal differentiation, and promotes alveolar differentiation.	[39, 50, 51]
Polycomb repressive complex 1 (PRC1)	PCGF	BMI1 or MEL18	H2AK119 mono-ubiquitylation	Repressive	BMI1 maintains stem/progenitor cells and restricts alveolar differentiation	[52]
	CBX	CBX2, CBX4, CBX6, CBX7, or CBX8				
	PHC	PHC1, PHC2, or PHC3				
	SCML	SCMH1 or SCML2				
	RING	RING2 or RING1				
Lysine demethylase (KDM) proteins	JARID1B/KDM5B/PLU-1		H3K4Me3 demethylation	Repressive and activating	GATA3 cofactor, promotes luminal differentiation	[55, 57–59]
	JMJD2B/KDM4B		H3K9Me3 demethylation	Activating	ERα cofactor, promotes luminal differentiation	[61]
	KDM6A/UTX		H3K27Me3 demethylation	Activating	Luminal transcription factor co-factor, promotes luminal differentiation	[62]
Plant homeodomain (PHD)-containing proteins	Pygo2		Binds to H3K4Me3	Not applicable	β-catenin co-factor, maintains stem/progenitor cells by restricting Notch-mediated luminal differentiation	[65]
Bromodomain and extra-terminal domain (BET) proteins	BRD4		Binds to acetylated lysines on histones	Activating	FOXO1 cofactor, maintains basal epithelial phenotype	[76]

## Histone modifications

Histone modifications occur on lysine and arginine residues and regulate DNA accessibility, or act as protein docking sites for the initiation of downstream biological processes, including chromatin compaction, transcriptional regulation, and DNA repair [16]. Histone acetylation is associated with transcriptional activation [34], while histone methylation can either activate (trimethylation of lysine 4 on histone 3; designated as H3K4Me3) or repress (H3K27Me3 or H3K9Me3) transcription [35, 36]. Bivalent promoters, containing both active H3K4Me3 and inactive H3K27Me3 marks, are often found at the promoters of key developmental genes and signal a repressed yet poised state, which allows for rapid activation or silencing of genes during differentiation [37]. These were originally discovered in ES cells and have since been identified in adult stem cells. Histones flanking active enhancer regions are marked by H3K4Me1 and H3K27ac modifications [38]. Histone modifications are most effectively mapped using chromatin immunoprecipitation followed by DNA sequencing (ChIP-Seq).

### Rewriting the histone code through mammary gland development

Mammary subpopulations and regulatory elements governing transcription have been defined through the integration of ChIP-Seq data with transcriptional signatures [28, 39–42]. The diverse chromatin states that distinguish between mammary epithelial subpopulations are as extensive as those that distinguish epithelial subpopulations from developmentally unrelated stromal cells [40]. The largest variations in chromatin state occurred in enhancer regions, although there were also significant variations in promoter regions [40]. This shows that the chromatin state of cell-specific regulatory elements is a key determinant of cell type, even within the same epithelial lineage.

Histone methylation changes correlate with gene expression changes during lineage restriction. In the mouse mammary gland, luminal progenitor-defining genes [43] have higher H3K4Me3 coverage and lower H3K27Me3 coverage in luminal progenitor cells compared with basal cells [39]. Genes within the mature luminal signature [43] show the same pattern when comparing mature luminal cells with luminal progenitors [39]. Histone modifications are also altered upon pregnancy. Luminal cells differentiate into milk-secreting alveolar cells in preparation for lactation, which results in decreased repressive marks and increased active marks in key luminal differentiation and milk-production genes. Similar results were found in sorted human mammary epithelial populations [28, 40]. Interestingly, the genes repressed in luminal and basal subsets are often present in large regions enriched for H3K27Me3 marks (K27 blocs), which may allow for cell-type specific co-ordinated gene silencing [28]. These studies demonstrate that distinct histone methylation profiles influence gene expression changes that direct basal to luminal progenitor differentiation and the maturation of luminal progenitors to luminal and alveolar cells (Fig. 1). Histone modifications also regulate gene expression within the heterogeneous basal population. A small population of quiescent MaSCs, marked by co-expression of LGR5 and TSPAN8, have recently been purified from the mouse mammary epithelium [44]. These cells share similarities with quiescent stem cells in other tissues and have distinct H3K4Me3 and H3K27Me3 landscapes compared with the other basal cells [44].

Analysis of bivalent promoters in the mammary epithelial subpopulations has led to some interesting discoveries. Luminal progenitor cells have intermediate promoter features between basal and mature luminal cells. For example, promoters of genes involved in basal functions (such as extracellular matrix organisation) are marked by H3K4Me3 in basal cells, are bivalent in luminal progenitors, and are marked with H3K27Me3 in mature luminal cells [40]. This corresponds with a decrease in gene expression [40]. This is consistent with the model that luminal progenitors are derived from basal cells. However, it is not possible to discern whether luminal commitment of basal cells happens in the adult gland or if it occurs during embryonic development and is maintained in the adult gland. While each subset contains bivalent domains, the highest number occurs in the terminally differentiated mature luminal subset [39, 40]. This challenges the dogma that bivalent domains operate predominantly in stem cells to restrict lineage-specific gene expression [37]. Instead, bivalent domains may be a more general phenomenon in cells of all differentiation states. Key developmental transcription factors within basal and luminal cells contain bivalent promoters; this may keep these genes poised, enabling rapid response to environmental stimuli [28, 40]. For example, in differentiated myoepithelial cells, the epithelial-mesenchymal transition (EMT) transcription factor ZEB1 is held in a bivalent state. Stimulation with transforming growth factor (TGF)β results in the removal of the repressive H3K27Me3 mark while maintaining the active H3K4Me3 mark. This leads to rapid transcription of ZEB1 and de-differentiation of the myoepithelial cells into stem-like cells in culture [12].

The following section will summarise the current literature surrounding the effector proteins mediating the epigenetic changes to the histone code.

### Polycomb group proteins maintain stem/progenitor cells

Polycomb group (PcG) proteins are epigenetic repressors that participate in the establishment and maintenance of cell identity. PcG proteins bind and repress genes that drive differentiation in embryonic and somatic stem cells. Differentiation is accompanied by loss of PcG binding and increased activation of PcG target genes [45]. In mammals there are two PcG chromatin-modifying complexes, Polycomb repressive complexes 1 and 2 (PRC1 and PRC2). These complexes work in a co-ordinated fashion to mediate repression [45]. The PRC2 complex is comprised of SUZ12, EED, RBBP4 or RBBP7, and EZH2 or EZH1 (Table 1). Initial recruitment of PRC2 to the chromatin depends on the DNA methylation state, pre-existing histone modifications, and recruitment by sequence-specific transcription factors [46]. EZH2 catalyses trimethylation of H3K27, which leads PRC1 recruitment. PRC1 is comprised of one member from each of the following paralog groups: PCGF, CBX, PHC, SCML, and RING [47] (Table 1). PRC1 ubiquitinates H2AK119, which represses transcription by condensing the chromatin and pausing RNA polymerase II [48].

PcG proteins are important for mammary gland development. As discussed above, H3K27 methylation is associated with gene expression changes that accompany mammary lineage commitment. EZH2, the catalytic subunit of PRC2, has been implicated in co-ordinating histone methylation changes during differentiation. Changes in EZH2 activity are regulated by progesterone during pregnancy and are mirrored by changes in global H3K27Me3 levels, coupling hormonal cues to changes in the epigenetic landscape [39]. Over-expression of EZH2 leads to multi-layered ducts and luminal cell hyperplasia, suggesting that EZH2 drives luminal expansion [49]. Conversely, EZH2 knock-out mice have delayed ductal elongation, and cells derived from these mice have a lower re-populating capacity in vivo and lower clonogenic activity in vitro. Doxycycline-inducible knock-out of EZH2 depletes the luminal progenitor pool, strengthening the role for EZH2 in maintaining luminal progenitors [50]. EZH2 supresses cell cycle inhibitors (e.g. *Ink4a* and *Cdkn1a*) and genes involved in epidermal differentiation, suggesting that EZH2 plays a critical role in progenitor cell proliferation and preventing activation of extraneous differentiation programmes [39]. Paradoxically, loss of EZH2 in the mammary epithelium did not alter H3K27Me3 ChIP-Seq profiles. However, because this was conducted on unsorted mammary tissue it is possible that stromal cells with intact EZH2 expression masked the true epigenetic modifications in the mammary epithelial cells. Another possibility is that EZH1, a weaker histone methyltransferase, compensates for EZH2 [51]. EZH2-deficient mice produce milk yet cannot support their pups. This is likely due to a combination of impaired progenitor proliferation leading to reduced alveolar unit density and impaired alveolar differentiation [39, 50].

BMI1, a member of the PCGF paralog group of the PRC1 complex, has also been implicated in mammary gland development. Like EZH2 knock-out mice, BMI1 knock-out mice have severely stunted ductal trees during pubertal development. Mammary epithelial cells from these mice have a 14-fold reduction in re-populating capacity upon serial transplantation, demonstrating the role of BMI1 in MaSC self-renewal [52]. BMI1 is also important for human MaSC self-renewal, where over-expression and knock-down of BMI1 increases and decreases mammosphere-forming capacity, respectively [53]. BMI1 also restricts alveolar differentiation; over-expression of BMI1 blocks alveolar differentiation and loss of BMI1 causes premature alveolar development [52]. The opposing roles of PcG proteins EZH2 and BMI1 in promoting and restricting alveolar differentiation, respectively, is an unexpected result that warrants further investigation.

### Lysine demethylases JARID1B, JMJ2B, and KDM6A drive luminal fate commitment

While histone methylation is well studied, enzymes that remove these marks, histone demethylases (known as KDMs), have only been identified more recently. Before their discovery, it was thought that histone methylation was an irreversible modification. It is now evident that histone demethylases play pivotal roles in modifying histones to determine whether a cell maintains multipotency or differentiates. As well as modifying the histone tails directly, certain family members can recruit PcG proteins to further modify chromatin [45]. There are six families of histone demethylase proteins, KDM1–6, each with multiple members that have distinct substrate specificity [54]. Members of the KDM2–6 families contain a Jumonji (or JmjC) domain, which uses a demethylation mechanism distinct from KDM1. So far, three JmjC domain-containing proteins have been identified as important regulators of mammary gland development: JARID1B (KDM5B, PLU-1), JMJ2B (KDM4B), and KDM6A (UTX) (Table 1).

JARID1B removes tri- and di-methylation of H3K4 and is thought to repress transcription [55, 56]. Complete and functional JARID1B knock-out mice have defects in pubertal mammary gland development, including a reduced number of TEBs, less side branching, and impaired ductal elongation [57, 58]. JARID1B mRNA is expressed in both murine basal and luminal lineages (Holliday et al., unpublished data) and seems to be important for rapid epithelial cell proliferation during puberty but not in alveolar development during pregnancy since JARID1B knock-out mice are able to produce milk [58]. The mammary developmental defects in these mice are partially due to a deficiency in systemic oestrogen levels; however, JARID1B also has a mammary cell-intrinsic function [58]. Gene expression analysis on primary mammary epithelial cells and cell lines with perturbed JARID1B expression revealed that JARID1B promotes luminal lineage-specific gene expression and represses basal-specific genes [56, 58, 59]. Key luminal lineage commitment genes (*Elf5*, *Esr1*, *Pgr*, *Prlr*, and *Stat5a*) are downregulated in the JARID1B-deficient mammary epithelial cells and breast cancer cell lines [58, 59]. In some but not all cases, increased gene expression was a consequence of JARID1B binding directly to chromatin, overlapping with active H3K4Me2/3 modifications. This is counter-intuitive given its histone demethylase activity on H3K4. It has been suggested that the PHD domain of JARID1B mediates recognition and binding to H3K4Me3 marks, leading to fine-tuning of H3K4 methylation [59, 60]. Zou et al. [58] observed co-binding of JARID1B and the luminal transcription factor GATA3 at the *Foxa1* and *Stat5a* promoters and loss of GATA3 binding in JARID1B knock-out mammary epithelial cells, arguing that JARID1B and GATA3 can act co-operatively to mediate transcription. JARID1B has also been shown to interact with oestrogen receptor (ER)α in the COS-7 cell line [57]. However, this has not been validated in mammary epithelial cells.

Trimethylation of H3K9 and H3K27 is associated with an inactive chromatin state. JMJD3B and KDM6A demethylate H3K9 and H3K27, respectively, and are therefore transcriptional activators [54]. Knock-out of either of these proteins results in defects in pubertal mammary gland development [61, 62]. JMJD2B interacts with ERα and oestrogen stimulation causes JMJD2B and ERα to localise to chromatin and demethylate H3K9 at ERα target genes [61]. KDM6A knock-out luminal mammary epithelial cells have a gene expression signature more similar to wild-type basal cells than wild-type luminal cells [62]. Like JMJD2B, KDM6A may also be a co-factor for luminal transcription factors since ChIP-Seq analysis on whole mammary glands reveals that KDM6A could bind to promoters and enhancers of ERα, progesterone receptor (PR), and ELF5 target genes [62]. Paradoxically, H3K27Me3 marks were unchanged upon KDM6A knock-out, suggesting that KDM6A has histone demethylase-independent functions, or that KDM6B can compensate for KDM6A loss [62].

### Pygo2 maintains mammary stem cells

The chromatin binding protein Pygo2 is part of the Pygopus family of proteins, which contain a highly conserved PHD domain. Pygo2 is an essential component of the Wnt/β-catenin signalling pathway [63], fundamental for maintaining stem cell self-renewal in many tissues including the mammary gland [8]. Unlike the PcG and the KDM proteins, Pygo2 does not directly modify chromatin; instead it recognises and binds active H3K4Me3 modifications via its PHD domain. Basal-specific deletion of Pygo2 results in a two-fold reduction of the basal population, decreased the re-populating capacity, and a basal cell gene expression profile which more closely resembles a luminal signature than a MaSC/basal signature [64, 65]. Components of the Notch signalling pathway, a key driver of luminal cell differentiation [66], are upregulated in Pygo2-deficient basal cells suggesting that Pygo2 normally acts to repress Notch signalling. Pygo2 is required for recruiting β-catenin to the Notch3 locus and maintaining the Notch3 gene in a bivalent state, such that loss of Pygo2 permits Notch-mediated luminal differentiation [65]. Taken together, these studies highlight the role of Pygo2 as a Wnt/β-catenin co-factor that maintains the basal fate by suppression of Notch signalling.

## Relevance to breast cancer

There is increasing evidence that different breast cancer subtypes arise from distinct developmental stages along the differentiation hierarchy and retain characteristics of their cell of origin [67]. The epigenetic processes that determine cell fate in normal cells are often hijacked by cancer cells [20, 68]. A comprehensive review of epigenetic perturbation in breast cancer is beyond the scope of this review, although several key examples of developmental epigenetic mechanisms gone awry in breast cancer are discussed below.

The genomes of cancer cells are globally hypomethylated compared with normal cells, resulting in genomic instability [69]. Cancer cells also harbour selective hypermethylation of promoter CpG islands in tumour suppressor genes [69, 70]. Consistent with the role of DNMT1 in maintaining MaSCs, it is also required for cancer stem cell (CSC) maintenance in the MMTV-Neu-Tg mouse mammary tumour model of HER2^+^ breast cancer [33, 71]. DNMT1 is highly expressed in breast CSCs where it methylates and silences several tumour suppressor genes including *Isl1*, whose gene product inhibits ERα-mediated transcription [33]. Treatment of mice with the clinical DNMT inhibitor 5-AzaC reduces the CSC pool and significantly improves survival, especially when combined with a histone deacetylase (HDAC) inhibitor [71].

In addition to DNA methylation, cancer cells also display perturbation of histone modifiers [20]. For example, in non-malignant cells, the core PRC2 component, EZH2, is required for the proliferation of progenitor cells [39, 51]. EZH2 is over-expressed in high-grade basal-like breast cancer where it likely plays a similar role [72]. Similarly, the PRC1 protein BMI1, whose role is to sustain self-renewal of normal mammary stem cells [52, 53], also has proto-oncogenic functions in breast cancer. BMI1 is over-expressed in aggressive basal-like breast cancers where it promotes EMT and self-renewal and is thought to confer drug resistance [73, 74]. JARID1B normally promotes luminal differentiation [58] and is frequently amplified and over-expressed in luminal breast cancer [59]. In this context, JARID1B is a highly active luminal lineage-specific proto-oncogene that correlates with poor patient outcome [59]. These examples highlight the need to understand the context-dependent regulation of these epigenetic factors in normal development.

Unlike genetic aberrations, epigenetic modifications are reversible, making epigenetic therapy an attractive avenue for treatment. Bromodomain and extra-terminal (BET) domain inhibitors are currently under clinical trial for the treatment of various cancers [75]. These drugs target proteins with a bromodomain, which recognises acetylated histone marks. Bromodomain-containing protein 4 (BRD4) co-operates with FOXO1 to promote the expression of basal genes in MCF10A normal mammary epithelial cells [76]. BET domain inhibitors are generally more efficacious in triple-negative breast cancer (TNBC) cell lines compared with luminal and HER2^+^ breast cancer cell lines [77]. Interestingly, these drugs seem to push the TNBC cells to a more differentiated luminal state [77].

Breast tumours are not only made up of epithelial cells, but also contain a heterogeneous microenvironment encompassing multiple different cell types including cancer-associated fibroblasts, dendritic cells, macrophages, and lymphocytes [78]. Epigenetic drugs do not only target the epithelial cells intrinsically but can also target the cross-talk between the epithelial cells and stromal cells. It has recently been shown that epigenetic therapies can sensitise cancer cells to the host immune system and boost the effects of immunotherapies such as check-point inhibitors (reviewed in [79]). This is particularly relevant to breast cancer, where treatment with check-point inhibitors has been shown to have limited efficacy compared with other cancer types [80].

Studying the context-dependent role of epigenetic modifiers in development has shaped our understanding of their role in different breast cancer subtypes. This research has uncovered exciting potential novel therapeutic targets and this list is continuing to grow.

## Conclusions

In response to microenvironmental cues, multiple layers of epigenetic regulation work in concert to maintain the undifferentiated MaSC state, or to direct the differentiation into specialised myoepithelial, luminal, and alveolar cells (Fig. 1). This review has focused on DNA methylation and the histone code but did not include nucleosome positioning and the three-dimensional organisation of chromatin within the nucleus. Systematic studies of genome-wide chromatin remodelling through mammary gland development and cell fate decisions are lacking and present an exciting area for further investigation.

Perturbation of epigenetic mechanisms can lead to the onset of different subtypes of cancer in a highly lineage-specific manner. A rigorous understanding of the epigenetic processes governing normal mammary development is central to our understanding of breast cancer aetiology and also for employing epigenetic therapies, which are becoming more commonly used in cancer treatment.

There is an increasing appreciation that differentiation occurs as a continuous spectrum, rather than proceeding through stable populations of cells with discrete identities [81]. This presents a challenge when studying bulk cell populations, as do the majority of studies described in this review. Technical advancement in this space has made it possible to perform single-cell ChIP-Seq, RNA-Seq, and the newly developed ATAC-Seq to decipher regions of open and closed chromatin [82]. Future studies will employ these cutting-edge technologies to generate chromatin maps and gene expression profiles to better understand the epigenomic and transcriptomic events that accompany lineage commitment at cellular resolution.

## Abbreviations

5-AzaC: 5-Azacitidine
ATAC: Assay for transposase-accessible chromatin
BET: Bromodomain and extra-terminal
ChIP: Chromatin immunoprecipitation
CSC: Cancer stem cell
DNMT: DNA methyltransferase
EMT: Epithelial-mesenchymal transition
ER: Oestrogen receptor
ES: Embryonic stem
HDAC: Histone deacetylase
KDM: Lysine demethylase
MaSC: Mammary stem cell
PcG: Polycomb group
PR: Progesterone receptor
PRC: Polycomb repressive complex
TEB: Terminal end bud
Tet: Ten-eleven translocation
TNBC: Triple-negative breast cancer
